# Risk Score for Prediction of Acute Kidney Injury in Patients with Acute ST-Segment Elevation Myocardial Infarction

**DOI:** 10.1155/2022/7493690

**Published:** 2022-12-20

**Authors:** Hui Zhao, Runran Miao, Fei Lin, Guoan Zhao

**Affiliations:** ^1^First Affiliated Hospital of Henan Polytechnic University, Jiaozuo, Henan 454000, China; ^2^Heart Center of First Affiliated Hospital of Xinxiang Medical University, Xinxiang, Henan 453100, China; ^3^Henan Province Cardiovascular Disease Clinical Data and Sample Resource Bank Engineering Research Center, Xinxiang, Henan 453100, China

## Abstract

**Background:**

Acute kidney injury (AKI) is an important comorbidity of ST-Segment Elevation Myocardial Infarction (STEMI) and worsens the prognosis. The purpose of this study was to investigate the relationship between clinical data, test results, surgical findings, and AKI in STEMI patients and to develop a simple, practical model for predicting the risk of AKI.

**Method:**

Prognostic prediction research with clinical risk score development was conducted. The data used for model development was derived from the database of the Henan Province Cardiovascular Disease Clinical Data and Sample Resource Bank Engineering Research Center. The data used for external validation was derived from the China Chest Pain Center database. The study endpoint was defined as the occurrence of AKI. Logistic regression analysis was used to identify independent predictors of AKI. Logistic coefficients of each predictor were used for score weighting and transformation. The predictive performance of the newly derived risk scores was validated, respectively, by receiver operating characteristic (ROC) regression in the development population and the external validation population.

**Result:**

A total of 364 patients, 57 (15.6%) with AKI and 307 (84.4%) without AKI, were included for score derivation. The validation crowd includes 88 STEMI patients in different institutions. A total of 11 potential predictors were explored under the multivariable logistic regression model. The new risk score was based on five final predictors which were age > 72 years, ejection fraction of no more than 40%, baseline serum creatinine > 102.7 mmol/L, red blood cell distribution width > 13.15, and culprit lesion located in the middistal segment. With only five predictor variables, the score predicted the risk of AKI with the effective discriminative ability (area under the receiver operating characteristic curve (AuROC): 0.721, 95% confidence interval (CI): 0.652-0.790). In the external validation, the newly developed score confirmed a similar discrimination as the crowd used for derivation (AuROC: 0.731, 95% CI: 0.624-0.838).

**Conclusion:**

The newly developed score was proved to have good predictive performance and could be practically applied for risk stratification of AKI in STEMI patients.

## 1. Introduction

ST-Segment Elevation Myocardial Infarction (STEMI) is an acute, life-threatening state of coronary heart disease and can lead to a variety of complications that may worsen the prognosis of STEMI [1]. Acute kidney injury (AKI) is a common syndrome manifested by an increase in blood creatinine and a decrease in glomerular filtration rate (GFR), which can occur secondary to STEMI [2]. Blood creatinine and GFR have been shown to be predictors of short- and long-term mortality in STEMI patients and used in risk scores [3]. As the reflection of the patient's renal and cardiac functional reserve, AKI is an important complication and an independent predictor of long-term mortality in patients with STEMI [4, 5]. The occurrence of AKI complicates the condition of STEMI patients which leads to prolonged hospital stay and increased mortality during hospitalization and cased poor short-term and long-term outcomes finally [6–8]. Identifying valid and simple predictors can help clinicians to identify high-risk patients early for early intervention. In previous studies, several factors have been shown to be predictive of the development of AKI, such as age, left ventricular ejection fraction, and inflammatory factors, and have provided assistance in clinical treatment. The composite factor generated from the combination of the underlying factors has better predictive performance because multiple underlying factors' predictive power is calculated simultaneously. For example, the ratio of platelet to lymphocyte has an ability to predict the occurrence of contrast-induced AKI in STEMI patients undergoing primary percutaneous coronary intervention [9]. In this study, we investigated possible predictors of AKI by analysing various clinical data of STEMI patients and the correlation with the occurrence of AKI and developed a predictive model in a simple and easy-to-use manner.

## 2. Materials and Methods

### 2.1. Selection of Participants

The study participants' data used for prediction model development were derived from the database of the Henan Province Cardiovascular Disease Clinical Data and Sample Resource Bank Engineering Research Center and included a total of patients aged 18 years or older with acute STEMI treated at the First Affiliated Hospital of Xinxiang Medical College from July 2018 to April 2021. Patients with a history of previous renal failure, patients without primary coronary angiography, and patients with incomplete vital information (such as lack of multiple serum creatinine tests to determine the occurrence of AKI) were excluded from the study. The data used for external validation were derived from patients with acute STEMI admitted to the First Affiliated Hospital of Henan Polytechnic University from June 2021 to December 2021, which were also registered in the China Chest Pain Center database and the date can be browsed on the Internet.

### 2.2. Data Collection and Definition

Clinical characteristics and potential predictors were extracted from routinely collected medical records including age, gender, blood pressure at admission, and risk factors for atherosclerotic such as hypertension, diabetes mellitus, profile of blood lipid, and cholesterol. AKI was defined as a rise in serum creatinine of more than 26.5 *μ*mg/L within 48 hours of admission or a rise in serum creatinine of more than 50% of the baseline level within 1 week, as referenced in the latest KDIGO criteria developed by the Kidney Disease: Improving Global Outcomes (KDIGO) [10]. The diagnostic criteria for STEMI were based on the guidelines of the European Society of Cardiology (ESC) [11]. Hypotension was defined as a systolic blood pressure of less than 90 mmHg on admission. Heart failure was defined as Class III and IV in the Killip classification of myocardial infarction.

All participants received primary coronary angiography within 24 hours of admission and their vascular lesions, location of the culprit lesion, and whether they were treated with PCI had been recorded. Coronary arteries were segmented according to the American Heart Association and Society for Cardiovascular Computed Tomography criteria to distinguish culprit lesions located in the proximal or middistal segments [12]. The segment after the midpoint of the origin to the turn in the right coronary artery and the segment after the first major branch in the left anterior descending branch and the left circumflex branch was defined as the middle and distal segment. Culprit lesions located in the proximal segment were called proximal culprit lesions while those in the middle and distal segments were called middistal segment culprit lesions (MDCLs).

### 2.3. Ethical Review and Informed Consent

This study was conducted in accordance with the principles of the Declaration of Helsinki. The Henan Province Cardiovascular Disease Clinical Data and Sample Resource Bank Engineering Research Center and the China Chest Pain Center database used in this study are publicly available databases. All patient information was completely anonymized to the investigators, and the study data were used only for anonymous retrospective analysis. Therefore, the need for an ethical approval statement and informed consent was waived for this manuscript.

### 2.4. Sample Size Calculation and Statistical Methods

Since there is no universally recommended sample size calculation method for clinical risk scoring models, this study used all available data in the database for the derivation of scoring models to maximize the accuracy of the scores.

All statistical analysis operations and procedures were performed using the SPSS 25.0 computer package. Continuous variables with normal distribution were expressed as mean standard deviation and analysed by Student's *t*-test; continuous variables with nonnormal distribution were expressed as quartile method and analysed by Mann–Whitney test. Categorical variables were expressed as percentages and analysed using the chi-square test or Fisher's exact test. Logistic regression analysis was used to analyse the predictive effect of variables and to determine scoring models. The predictive performance of the newly derived risk scoring models was validated, respectively, by receiver operating characteristic (ROC) regression in the development population and the external validation population. A *P* value of <0.05 was considered to indicate statistical significance.

### 2.5. Model Development

With reference to the results of previous studies on AKI and the types of available data, the patient's clinical data, laboratory tests, and surgery-related content were selected for the determination of initial factors. A preliminary analysis of all potential predictors was performed using logistic regression analysis and receiver operating characteristic (ROC) analysis. The hazard ratio (OR), *P* value, and area under the ROC curve with its 95% confidence interval (AuROC, 95% CI) were reported separately for each potential predictor. Continuous variables were obtained by ROC analysis to obtain the best cutoff point and this value was reduced to binary variables, in order to simplify the model. Multivariate logistic regression analysis was performed, and predictors with *P* values >0.1 were sequentially eliminated from the logistic regression model to determine the final predictors of AKI and the associated regression coefficients. Each final predictor was assigned with specific score based on each item's logistic regression coefficient. The logistic coefficient of each predictor was divided by the lowest coefficient in the model and subsequently rounded up to the nearest no decimal integer for forecasted applicability, while the predictor with the smallest regression coefficient was defined as 1 point.

### 2.6. Validation of the Model

The discriminability and calibration of the predictive models were assessed by ROC analysis. The Hosmer-Lemeshow test assessed the fit of the model. The observed risk of developing AKI was compared with the risk predicted by the new model and a calibration chart was formed. The prediction model was tested by ROC analysis of external validation population and Hosmer-Lemeshow test to evaluate the predictive effect of the model on the external population.

## 3. Results

### 3.1. Participants

A total of 364 patients, 57 with AKI and 307 without AKI, were included for analysis. The incidence of AKI in the study cohort was 15.6%. The patients' baseline clinical characteristics, laboratory tests, and coronary intervention findings were presented in Tables 1 and 2. When compared to those without AKI, patients with AKI were older (65.3 ± 12.6 vs. 61.0 ± 12.4 years, *P* = 0.017) had lower hemoglobin (129.8 ± 22.2 vs. 15.7 ± 18.1, *P* = 0.031), had a higher prevalence of diabetes (33.3% vs. 19.9%, *P* = 0.026), had a higher proportion of ejection fraction ≤ 40 (10.5% vs. 3.2%, *P* = 0.020), had higher red blood cell distribution width (RDW) (12.91 ± 1.53 vs. 12.39 ± 0.98, *P* = 0.005), and had a higher proportion of culprit lesions located in the middistal segment (75.4% vs. 49.5%, *P* < 0.001). In this study, none of the patients required emergency hemodialysis.

### 3.2. Predictor Simplification

Hemoglobin, age, baseline serum creatinine, and red blood cell distribution width from Tables 1 and 2 were potential predictors. For simplification, the continuous variables were converted into dichotomous variables through the optimal cutoff point obtained from the ROC analysis results (Table 3).

These new variables were to univariate logistic regression analysis. Eleven potential clinical predictors were simultaneously explored under multivariable logistic regression (Table 4).

After sequential elimination of noncontributive and nonsignificant predictors, by using backward elimination (likelihood ratio, *P* < 0.100), five independent predictors were left in the final logistic model, including ejection fraction of no more than 40%, age > 72 years, creatinine > 102.7 mmol/L, RDW > 13.15, and middistal segment culprit lesions. The logistic coefficient of each predictor was used as a weight for score transformation (Table 5).

**Figure 1:**
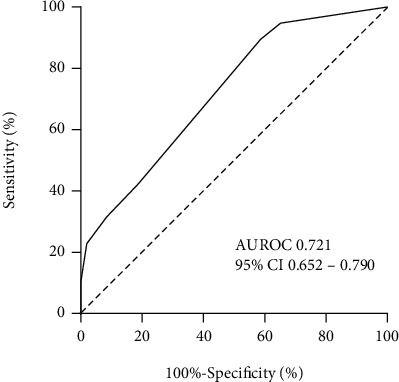
Receiver operating characteristic curve (ROC) of XMU score in discriminating AKI cases. AKI: acute kidney injury; XMU: Xinxiang Medical University.

The newly derived risk hierarchical fraction is named Xinxiang Medical University (XMU) score. XMU score ranged from a minimum of 0 to a maximum of 9 (Table 5). The score could predict the risk of AKI with good discriminative ability (AuROC: 0.721, 95% CI: 0.652-0.790) (Figure 1).

### 3.3. Calibration of Score

**Figure 2:**
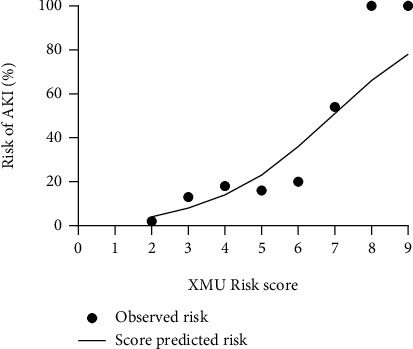
The observed incidence of AKI and the predicted incidence of AKI for different XUM fractions. AKI: acute kidney injury; XMU: Xinxiang Medical University.

**Figure 3:**
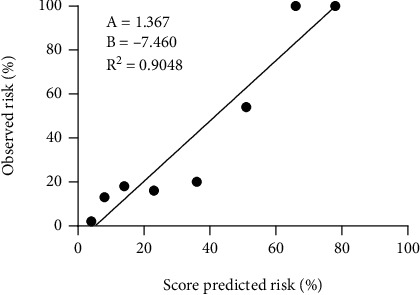
Linear regression analysis of the observed incidence of AKI and the predicted incidence of AKI. AKI: acute kidney injury.

The calibration of the score passed Hosmer-Lemeshow test, and the *χ*^2^ value was 0.110 (*P* = 0.190). The incidence of AKI predicted by the score and the actual incidence observed was calculated in the case of grouping according to the score (Figure 2). A linear regression analysis was also performed for both incidences, showing *R*^2^ = 0.9048, *P* < 0.001 (Figure 3). The predicted incidence fit with the observed incidence well.

### 3.4. External Validation

88 consecutive patients operated at the First Affiliated Hospital of Henan Polytechnic University was assessed with the XMU score. ROC analysis results showed that XMU score could predict the risk of AKI in external validation participants (AuROC: 0.731, 95% CI: 0.624-0.838). The Hosmer-Lemeshow test demonstrated a good overall calibration, with a *χ*^2^ of 4.589 (*P* = 0.332).

## 4. Discussion

This study analysed the relationship between AKI in STEMI patients and multiple clinical data and procedural findings, summarized the predictors of AKI, and derived simple predictive models (XMU score). XMU score has good distinction and calibration in the development participants and external verification participants. In this study, the ejection fraction value, age, baseline creatinine level, red blood cell distribution width, and coronary culprit lesion location were found to be related to the risk of AKI.

Ejection fraction value, age, and baseline creatinine level have been shown to be AKI risk predictive factors in previous studies [13]. The ejection fraction value can reflect the function of cardiac pump, and the decline in heart pump directly reduces the blood perfusion of the kidney, resulting in kidney dysfunction. Age is a confirmed AKI independent risk factor and was used in clinical predictive scoring models [13, 14]. Baseline creatinine levels in multiple AKI risk predictions were previously used as risk factors and showed predictive capabilities. Patients with high baseline creatinine were more likely to suffer AKI [15, 16]. Ejection fraction value, age, and creatinine also shown a relationship with long-term and short-term mortality in STEMI patients [17, 18]. The three predictors were continuous variables; taking into account the ease of use of the study requirements, the continuous variables were converted to the binary variables according to the critical value of ROC analysis and were included in the predictive model as the final predictive factor.

RDW reflects the degree of heterogeneity of red blood cell volume in peripheral blood and it was previously used in the differential diagnosis of anemia [19]. In this study, the risk of AKI was found to increase with increasing levels of RDW. Based on the results of ROC analysis, RDW > 13.15 was included in the prediction model as the final predictor variable. RDW's predictive ability of AKI may be related to inflammatory response. Recent results in recent years indicate that RDW is related to inflammatory response, oxidative stress responses to [20, 21], and inflammation and oxidative stress have been confirmed to be a risk factor of AKI.

This study found an increased risk of AKI in patients whose coronary culprit lesion occurred in the middle and distal segments. The exact cause cannot be determined. Rupture of atherosclerotic plaques within the coronary arteries was currently considered to be an important factor in the development of STEMI. Atherosclerotic plaque composition includes a necrotic core composed of lipids, necrotic material, cholesterol crystals, macrophages, foam cells, and a fibrous cap [22]. Plaques with a high percentage of necrotic core and less fibrous tissue are called Thin Cap Fibroatheroma (TCFA) [23]. Compared with plaques with thick fibrous caps, more plaque contents are released into the circulation during TCFA rupture, including inflammatory factors, cholesterol crystals, and necrotic material [24]. And these contents are risk factors for AKI [25, 26]. TCFA has been shown mainly concentrated in the middle and distal segments of the anterior descending and gyral branches [23, 27]. With intravascular ultrasound, Chung et al. found the correlation between plaque characteristics and plaque rupture location that plaques occurring in the middle segment of the coronary artery have a larger necrotic core than the proximal segment [28]. STEMI caused by TCFA rupture had a higher risk of AKI via more core contents being released, while MDSCL represented a high chance of TCFA rupture. Therefore, the predictive power of culprit lesion location for AKI was related to TCFA plaque distribution.

There are some limitations, such as small number of centers and small sample size; the study design was a retrospective analysis and lacked a rigorous prospective study. A larger prospective study should be conducted in the future to refine this study.

## 5. Conclusions

In conclusion, this study proposed the new risk score based on five independent predictors. The XMU score was proved to have good predictive value with fewer numbers of predictors and was shown to be a good predictor of the risk of AKI in STEMI patients, and externally validated. It can be used as a reference in clinical work.

## Figures and Tables

**Table 1:** Clinical characteristics.

Clinical characteristics	AKI (*n* = 57) (%)	Without AKI (*n* = 307) (%)	OR	*P*	AuROC (95% CI)
Age, years (mean ± SD)	65.3 ± 12.6	61.0 ± 12.4	1.029	0.017	0.590 (0.505-0.675)
Male	41 (71.9)	226 (73.6)	1.089	0.792	0.508 (0.426-0.590)
Hypertension	36 (63.2)	169 (55.0)	1.400	0.258	0.540 (0.459-0.591)
Diabetes	19 (33.3)	61 (19.9)	2.016	0.026	0.567 (0.483-0651)
Anemia	22 (38.6)	85 (27.7)	1.642	0.099	0.554 (0.471-0.637)
Heart failure	10 (17.5)	29 (9.4)	2.040	0.074	0.540 (0.459-0.620)
Hypotension	3 (5.3)	7 (2.3)	2.373	0.221	0.554 (0.471-0.637)
SBP (mmHg) (mean ± SD)	129.0 ± 26.3	127.2 ± 23.4	1.003	0.611	0.536 (0.448-0.623)
DBP (mmHg) (mean ± SD)	79.5 ± 15.7	91.0 ± 15.8	0.994	0.526	0.477 (0.394-0.560)
HR (bpm) (mean ± SD)	81.0 ± 15.8	80.1 ± 15.6	1.002	0.820	0.510 (0.429-0.591)
Ejection fraction (%)	53.0 ± 9.5	55.2 ± 7.4	0.966	0.053	0.430 (0.345-0.515)
≤50%	18 (31.6)	66 (21.5)	1.685	0.100	0.550 (0.466-0.634)
≤45%	9 (15.8)	25 (8.1)	2.115	0.074	0.538 (0.454-0.623)
≤40%	6 (10.5)	10 (3.2)	3.494	0.020	0.536 (0.451-0.621)
Laboratory values (mean ± SD)					
Creatinine (mmol/L)	69.0 ± 37.1	62.7 ± 25.3	1.007	0.126	0.501 (0.416-0.587)
Urea nitrogen (mmol/L)	6.22 ± 2.19	5.79 ± 1.87	1.112	0.127	0.539 (0.455-0.624)
Uric acid (mmol/L)	307 ± 91	311 ± 93	1.000	0.787	0.487 (0.405-0.569)
Hemoglobin (g/L)	129.8 ± 22.2	15.7 ± 18.1	0.984	0.032	0.426 (0.338-0.513)
Triglyceride (mmol/L)	1.38 ± 0.82	1.36 ± 0.99	1.017	0.910	0.521 (0.436-0.607)
Cholesterol (mmol/L)	4.49 ± 1.15	4.53 ± 1.14	0.966	0.791	0.464 (0.409-0.584)
LDL (mmol/L)	2.63 ± 0.93	2.59 ± 0.80	1.062	0.729	0.497 (0.409-0.584)
HDL (mmol/L)	1.26 ± 0.38	1.22 ± 0.31	1.493	0.326	0.545 (0.468-0.622)
NLR (%)	10.44 ± 8.08	9.74 ± 8.16	1.010	0.554	0.535 (0.454-0.616)
RDW	12.91 ± 1.53	12.39 ± 0.98	1.403	0.005	0.627 (0.546-0.705)

AKI: acute kidney injury; SD: standard deviation; OR: odds ratio; AuROC: area under the receiver operating characteristic curve; SBP: systolic blood pressure; DBP: diastolic blood pressure; HR: heart rate; LDL: low-density lipoprotein; HDL: high-density lipoprotein; NLR: neutrophil-to-lymphocyte ratio; RDW: red blood cell distribution width.

**Table 2:** Operative procedure and findings.

Procedural findings	AKI (*n* = 57) (%)	Without AKI (*n* = 307) (%)	OR	*P*	AuROC (95% CI)
Single-vessel disease	23 (40.3)	60 (19.5)	0.672	0.330	0.472 (0.393-0.552)
Double-vessel disease	31 (54.3)	61 (19.9)	0.658	0.305	0.471 (0.391-0.550)
Triple-vessel disease	3 (5.3)	186 (60.6)	1.667	0.107	0.557 (0.478-0.636)
MDCL	43 (75.4)	152 (49.5)	3.132	<0.001	0.630 (0.555-0.705)
PCI treated	49 (86.0)	269 (87.6)	0.865	0.730	0.492 (0.409-0.574)

AKI: acute kidney injury; OR: odds ratio; AuROC: area under the receiver operating characteristic curve; CI: confidence interval; PCI: percutaneous coronary intervention; MDCLs: middistal segment culprit lesions.

**Table 3:** New predictor.

Continuous predictor	Cutoff value	New predictor	OR (95% CI)
Age (years)	72	Age > 72 years	2.558 (1.390-4.710)
Hemoglobin (g/L)	117.5	Hemoglobin < 117.5 g/L	3.199 (1.644-6.224)
Creatinine (mmol/L)	102.7	Creatinine > 102.7 mmol/L	5.569 (2.152-14.410)
RDW	13.15	RDW > 13.15	3.886 (2.021-7.470)

OR: odds ratio; CI: confidence interval; RDW: red blood cell distribution width.

**Table 4:** Multivariable logistic regression analysis.

Variables	OR	95 CI	*P*
Age	1.009	0.972-1.047	0.634
Diabetes mellitus	1.473	0.725-2.993	0.284
Heart failure	1.561	0.581-4.195	0.377
Anemia	0.698	0.249-1.955	0.494
Ejection fraction ≤ 45	1.211	0.365-14.313	0.790
Ejection fraction ≤ 40	2.285	0.365-14.313	0.790
Age > 72years	1.312	0.467-3.689	0.607
Hemoglobin < 117.5 g/L	2.097	0.624-7.051	0.231
Creatinine > 102.7 mmol/L	3.348	1.126-9.954	0.030
RDW > 13.15	2.497	1.124-5.551	0.025
MDCL	4.151	2.000-8.617	0.001

OR: odds ratio; CI: confidence interval; RDW: red blood cell distribution width; MDCLs: middistal segment culprit lesions.

**Table 5:** Best multivariable clinical predictors and assigned item scores.

Predictors	OR (95 CI)	*P*	*β*	Item scores
Age > 72years	1.896 (0.950-3.786)	0.070	0.640	1
MDCL	3.973 (1.953-8.081)	<0.001	1.380	2
Creatinine > 102.7 mmol/L	4.168 (1.484-11.708)	0.007	1.427	2
RDW > 13.15	2.917 (1.395-6.102)	0.004	1.071	2
Ejection fraction ≤ 40	3.492 (1.001-12.184)	0.050	1.250	2
Constant	0.011	<0.001	-4.525	

OR: odds ratio; CI: confidence interval; RDW: red blood cell distribution width; MDCLs: middistal segment culprit lesions.

## Data Availability

The data used to support the findings of this study are available from the corresponding author or first author upon request.
